# Contact-Map-Driven Exploration of Heterogeneous Protein-Folding Paths

**DOI:** 10.1021/acs.jctc.4c00878

**Published:** 2024-09-04

**Authors:** Ziad Fakhoury, Gabriele C. Sosso, Scott Habershon

**Affiliations:** Department of Chemistry, University of Warwick, Coventry CV4 7AL, U.K.

## Abstract

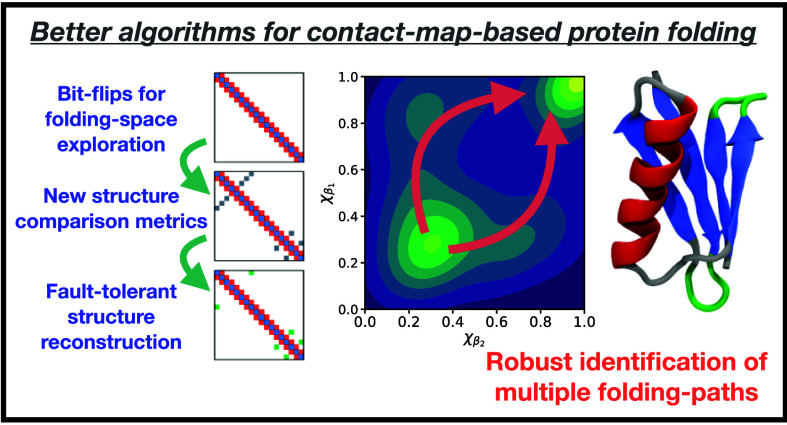

We have recently shown how physically realizable protein-folding pathways can be generated using directed walks in the space of inter-residue contact-maps; combined with a back-transformation to move from protein contact-maps to Cartesian coordinates, we have demonstrated how this approach can generate protein-folding trajectory ensembles without recourse to molecular dynamics. In this article, we demonstrate that this framework can be used to study a challenging protein-folding problem that is known to exhibit two different folding paths which were previously identified through molecular dynamics simulation at several different temperatures. From the viewpoint of protein-folding mechanism prediction, this particular problem is extremely challenging to address, specifically involving folding to an identical nontrivial compact native structure along distinct pathways defined by heterogeneous secondary structural elements. Here, we show how our previously reported contact-map-based protein-folding strategy can be significantly enhanced to enable accurate and robust prediction of heterogeneous folding paths by (i) introducing a novel topologically informed metric for comparing two protein contact maps, (ii) reformulating our graph-represented folding path generation, and (iii) introducing a new and more reliable structural back-mapping algorithm. These changes improve the reliability of generating structurally sound folding intermediates and dramatically decrease the number of physically irrelevant folding intermediates generated by our previous simulation strategy. Most importantly, we demonstrate how our enhanced folding algorithm can successfully identify the alternative folding mechanisms of a multifolding-pathway protein, in line with direct molecular dynamics simulations.

## 1 Introduction

Understanding how - and how quickly - a protein moves from an unfolded state to the native folded structure remains a grand challenge for computer simulations.^1^ Rapid advances in machine-learning (ML) strategies, particularly the development of AlphaFold2,^2^ have transformed the landscape in terms of our ability to predict the *final* folded structures of proteins, offering new routes to rapid in silico screening of drugs against novel protein targets. However, as well as folded structure, the dynamic sequence of events leading to formation of the native state is equally important in forming a holistic picture of protein functionality. For example, understanding protein folding dynamics is central to ongoing attempts to address diseases caused by protein misfolding and aggregation,^3^ while long-lived protein-folding intermediates represent a largely unexplored and often necessary source of new targets for traditionally “undruggable” proteins.^4^ Moreover, modern approaches to novel protein design would benefit from a measure of fold-ability in order to ensure verifiable function of any newly designed protein sequences.^5^

Previously,^6^ we have developed and demonstrated a new simulation strategy that aims to generate a folding-pathway ensemble without demanding extensive molecular dynamics (MD) simulations or predefined order parameters. Here, protein configurations are represented in the space spanned by the binary inter-residue contact-map, and we employ a simulated annealing (SA) optimization strategy to identify sequences of contact-map updates that definitively lead to formation of target folded states. For each directed-walk, we subsequently employ a back-mapping procedure, previously employed in the context of our research on automated chemical reaction discovery,^7−9^ to generate folding intermediate structures in Cartesian space. Using the sequence of intermediate structures, further analysis of the kinetics and thermodynamics associated with each elementary step of each folding sequence can be assessed using geometry optimization and nudged elastic band (NEB)^10^ calculations on an appropriate potential energy surfaces (PES).

To date, we have shown how this procedure can be readily used to fold model protein structures (described by an off-lattice HP model^11^) with up to 34 residues. Perhaps most importantly, we have also demonstrated that the folding pathways generated by our graph-driven sampling (GDS) strategy overlap with the folding ensemble generated by brute-force MD simulations. Specifically, multidimensional scaling (MDS) analysis based on a Fréchet distance between MD and GDS folding trajectories demonstrated that our discretized approach visits the same ensemble of intermediate folding structures as MD trajectories in the canonical ensemble.

In this paper, we tackle a more challenging protein-folding problem known to exhibit two distinct pathways - protein L/G. This protein exhibits a nontrivial compact native structure and folds through two distinct pathways that are associated with distinct secondary structural changes. Problems of this sort are of practical importance since it opens further doors to understanding and targeting protein intermediates from different mechanisms. The folding of protein L/G was previously studied using extensive coarse grained (CG) MD simulation using the so-called BLN PES.^12,13^ Despite the order of magnitude speed up associated with CG MD, attempts by Head-Gordon and co-workers to identify and characterize these two pathways still required extensive MD simulations at multiple temperatures to map out the free energy surface in terms of a few predefined order parameters. Moreover, if one were to use an all-atom model instead, this procedure would likely be infeasible due to the time-scales involved.

In our approach, we avoid integrating the system dynamics directly and therefore circumvent the associated timescale limitations. As described below, our GDS scheme does not scale with the time scale of the folding process, but rather with the number of mechanisms associated with the folding process. The generation of folding pathways in our GDS strategy is also unaffected by the physical PES model being employed; instead, the initial generation of folding paths in GDS is based solely on contact-maps, only requiring a physical interaction model to postevaluate the characteristics of each path once they have been generated. Furthermore, our GDS strategy does not rely on the a priori definition of order parameters, as is commonly employed many other accelerated MD schemes.

However, before deploying GDS to study heterogeneous protein-folding pathways identified for protein L/G, there remain a number of outstanding challenges in optimizing our directed-walk strategy as we seek to model the folding of larger, more complex proteins. The purpose of this article is to report on several algorithmic developments that dramatically improve the efficiency and reliability of our contact-map-based strategy to tackle the protein-folding problem; importantly, the developments highlighted here also provide the foundation to push toward applications to challenging atomistic simulations of protein-folding in the near-future.

In this paper, we specifically focus on three improvements of our GDS simulation approach. First, in seeking to identify the folding-path ensemble for larger protein structures we have found that the efficiency of our original directed-walk optimization strategy decreases. As described below, our current GDS approach represents folding pathways as sequences of discrete contact-map events, with the restriction that any single event represents either forming or breaking a *single* contact between residues. As the size of target protein structure increases, the number of events required to converge on the final folded structure similarly increases, meaning that increasingly long contact-map update sequences must be sought during SA optimization. Furthermore, the number of possible contact-map breaking or forming pairs that can be selected for a given event grows significantly with the size of the protein structure, and can lead to a large number of rejected steps during SA optimization. In this article, we show how this challenge can be addressed by modifying our framework for updating a proposed sequence of contact-map events.

A second challenge in GDS is finding a suitable metric to quantify the differences between protein structures based only on their contact-map representation, without the need for (relatively expensive) structural back-mapping. In our previous work using graph representations, we used the total number of edge differences to discriminate between the final contact-map generated by a given folding sequence and the target contact map. However, there are alternative methods to discriminate between two graphs that possess numerically preferable properties, such as permutational invariance.^7^ Furthermore, as discussed in this article, the number of contact differences between two contact-maps is generally not a good measure of structural “distance” when one additionally considers the back-mapped (Cartesian space) structures. To overcome this challenge, we also use this article to introduce a new contact-map-based metric that is compatible with our GDS approach for folding-path generation *and* better reflects structural similarity in the coordinate representation.

A third and final challenge to our existing GDS strategy arises in the back-mapping transformation from contact-maps to three-dimensional protein structures. When applied to larger protein structures, we found many of these transformations failed to find a three-dimensional protein structure that corresponded to a given contact map. We also note that the back-transformation procedure can fail when the structural restraints imposed by the contact-map are in conflict with each other. As such, this article will examine different proposed back-mapping strategies in order to determine a more effective algorithm.

The remainder of this article is structured as follows. In the Methods section, we briefly review our GDS approach, before describing and justifying the updates made in seeking to tackle more complex problems, such as identifying the heterogeneous folding paths of the L/G protein. In the Results and Discussion section, we explore how the three improvements to GDS described above impact the reliability and efficiency of our simulations and finally, in Conclusions, we highlight some possible future applications that will be enabled by these new developments.

## 2 Methods

In this section, we begin by introducing the folding problem for the L/G protein, following Head-Gordon and co-worker’s CG MD studies. We then summarize our previous approach to generating protein-folding pathways in contact-map space using GDS. Subsequently, we highlight the three new developments implemented in this article in order to improve the robustness, reliability and efficiency of GDS as we seek to study more complex protein-folding problems such as that embodied by the L/G protein system.

### 2.1 Protein Model and BLN Potential

**Figure 1:**
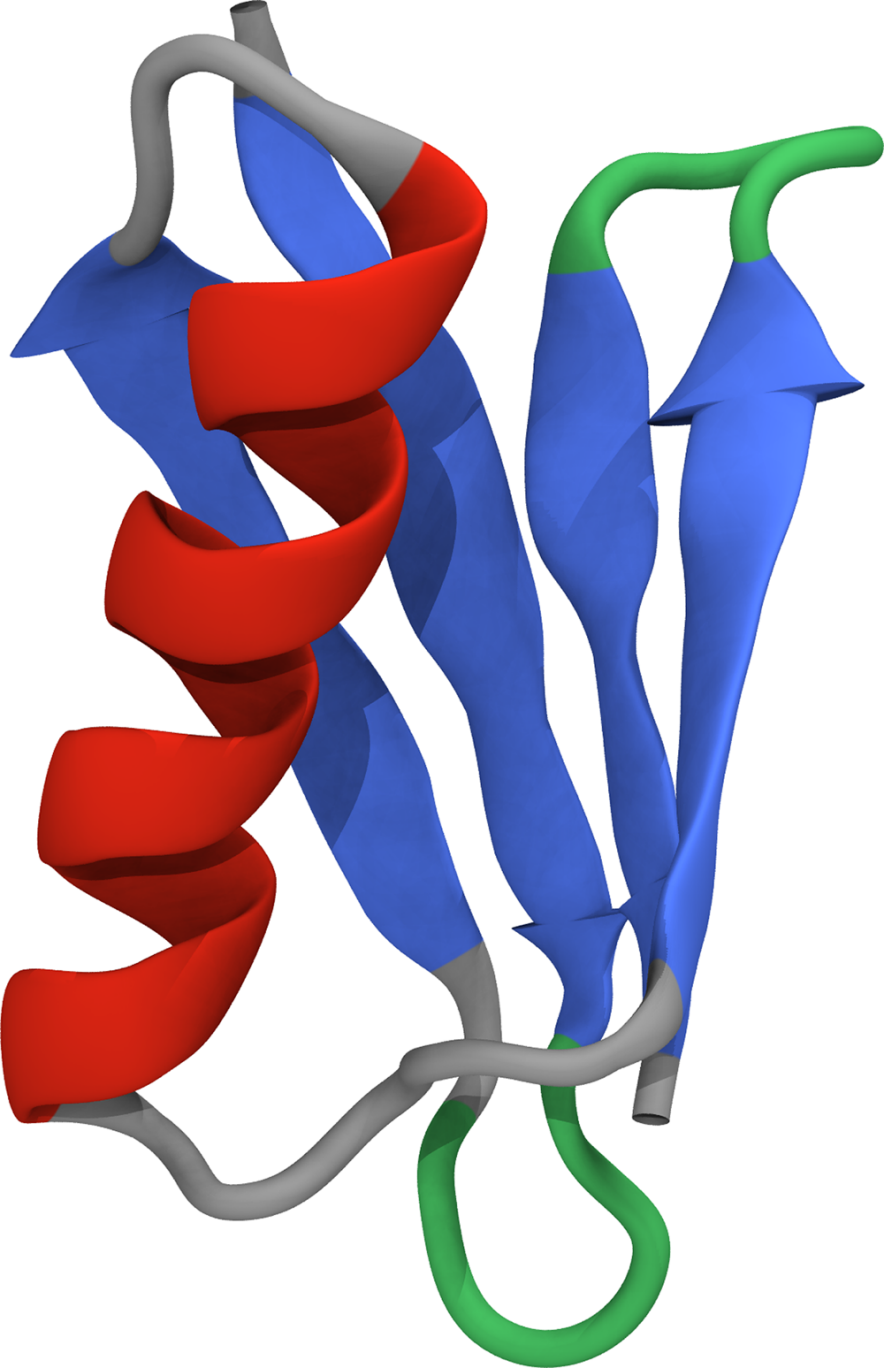
Ribbon visualization of the B1 domain of protein G (PDB 2GB1). The ribbon is colored according to secondary structure, illustrating the (red) α helix attached to (blue/green) beta hairpins, with gray and green representing short loop regions.

In their protein design work, Head-Gordon and co-workers designed a CG represented protein that folded into a structure that shared an identical folded topology with two Protein Domains: the B1 domain of protein L (PDB ID 2PTL) and the B1 domain of protein G (PDB ID 2GB1).^12^ Proteins L and G, although having identical folded structures, have different dominant folding pathways; the CG-designed protein was shown to fold through two pathways similar to the proteins L and G, respectively. This protein, named protein L/G, exhibits a nontrivial compact native structure with heterogeneous secondary structure elements (Figure 1). Through a free-energy histogram construction for protein L/G in the space of five order parameters, they uncovered the existence of two distinct mechanisms, corresponding to either protein L or G. The primary goal of this article is to boost the efficiency and robustness of our previous GDS strategy in order to enable the identification of these two folding paths - crucially *without* the need for direct MD simulations at different temperatures.

The CG potential employed here, and in the previous work of Head-Gordon, is referred to hereafter as the BLN model. Here, each amino-acid residue is represented as a single “bead” which is classified as being Hydrophobic (B), Hydrophilic (L) or Neutral (N). In addition, the BLN PES employs a secondary characterization of every dihedral angle (namely Helix [H], Extended Strand [E], Turn/Coil [T]), in order to bias the PES to reproduce expected secondary structure elements in the native structure.

In all of the following discussion, we use a set of reduced units. Here, the mass of each bead is defined to be 1 mu (mass unit), and the unit of energy ϵ is defined as the Lennard-Jones well-depth (at equilibrium distance) for interactions between hydrophobic residues. Furthermore, we employ Å as our distance unit, and time is therefore measured in units .

The BLN potential energy *V* (**r**) for a given configuration **r** is given by
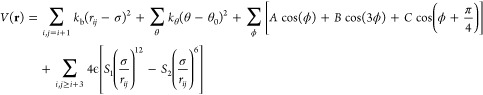
1where (θ, ϕ) are the bond angles and dihedral angles, respectively, and the first term is a sum over bonded-bead distances. A strong restraint is placed on consecutive bead pairwise distances, with *k*_b_ = 115.6 ϵ Å^–2^ and σ = 3.8 Å. A weaker restraint is placed on the angles, with *k*_θ_ = 10 ϵ rad^–2^ and θ_0_ = 1.8326 rad. In addition, {*A*, *B*, *C*, *S*_1_, *S*_2_} are bead-dependent parameters, where (*A*, *B*, *C*) are parametrized by the assigned secondary structure element (according to the target native structure), and (*S*_1_, *S*_2_) are determined according to the classification of the pair of beads in question as follows:
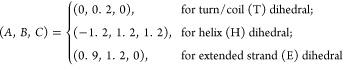

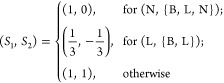
Here, (N, {B,L,N}) indicates interactions between N-labeled beads and any other bead-types, whereas (L, {B,L}) similarly represents interactions between L-type beads and either B- or L-labeled beads. We note here that the parametrization of the BLN model is defined by the native folded structure of the particular protein under investigation. However, the availability of previous MD simulations investigation of folding paths for protein L/G using this PES offers an important opportunity to employ GDS to study a multipathway folding system and drive the further improvements of our approach that are described below.

### 2.2 Initial GDS Methodology

Our motivation in previously introducing GDS was to use the inter-residue contact map (also interchangeably referred to here as the *graph* of the system) as a discretization of protein structure space. In the following, for an *N*-bead system, the contact-map **G** is an *N* × *N* square matrix that simply defines whether or not two residues in a protein structure are in close-contact according to a predefined distance criteria, as follows:
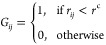
2Here, *r*_*ij*_ is the distance between residues (*i*, *j*) in the protein and *r*^c^ is an appropriately chosen cutoff distance. Our cutoff distance is typically chosen to be 8 Å, but a range of cutoffs from 7 to 11 Å have shown to be optimal dependent on the application.^14,15^ In our simulation approach, these differences have little overall effect because the structural reconstruction approach described later additionally employs an energy minimization that inevitably washes out these small differences.

Graph representations of structures have been employed in molecular rare event strategies with great success.^16−18^ More specifically for proteins, a contact map encodes secondary and tertiary structure information, which has proven to be a promising representation in several protein conformational dynamical applications.^19,20^ With this discretization, a protein-folding pathway can be represented as a sequence of “hops” between contact maps, where each consecutive hop represents an elementary conformational transition; we refer to this path representation as a graph sequence. Here, we briefly recount our initial strategy to find such graph sequences that - when back-transformed into a corresponding Cartesian-coordinate representation - proposed physically viable folding-pathways.

**Figure 2:**
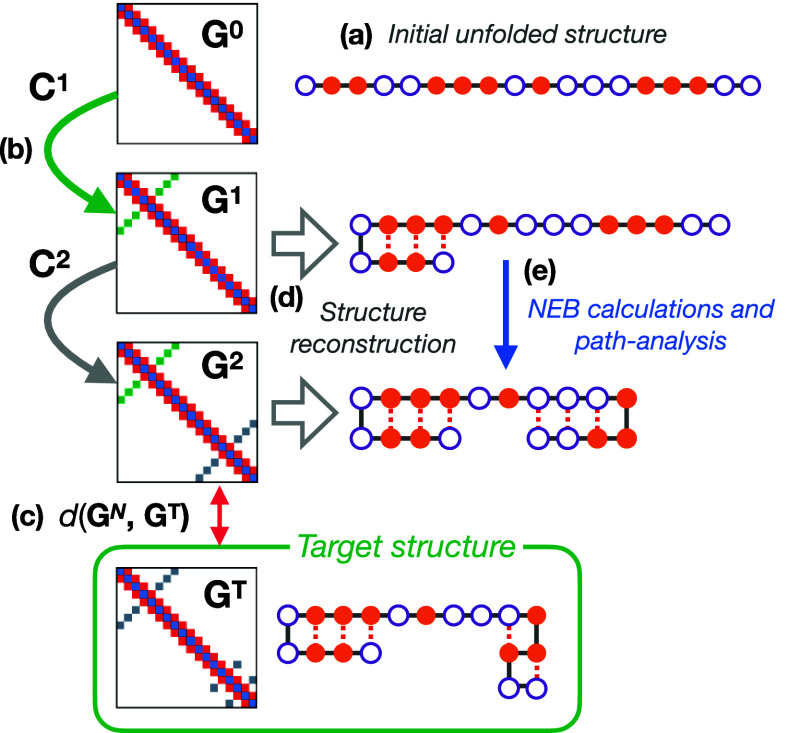
Schematics of the GDS strategy for folding-path generation. Starting from (a) an initially unfolded structure (with empty and filled circles representing arbitrary residue “beads”) we (b) propose a series of contact-map updates to generate a folding trajectory. The final generated contact-map is (c) subsequently compared to the target folded state. If further analysis is required, each intermediate contact-map is (d) back-transformed to Cartesian-space coordinates, before (e) further characterization of the folding pathway thermodynamics and kinetics is performed.

Our initial methodology (Figure 2) optimized the graph sequence [**G**^0^, **G**^1^,..., **G**^*N*^] by attempting to minimize a predefined discriminator function defining the “distance” between the final contact-map **G**^*N*^ and a target contact-map **G**^T^. Each graph-transition between **G**^*i*^ and **G**^*i*+1^ belongs to a predetermined set of transition moves (specifically, making a new inter-residue contact or breaking an existing inter-residue contact).

As described below, the discriminator function plays an important role in our GDS approach. In our initial report, we focused on determining folding paths for the off-lattice HP model,^11^ which defines each protein residue as a single hydrophobic or polar bead. This model was a simple phenomenological PES that is commonly used to showcase the hydrophobic collapse phenomena and, importantly, enabled ready generation of direct MD folding pathways to enable comparison to our graph-based approach. In the HP model, the folded state is often not a single unique structure, but rather any structure that shares the same set of hydrophobic coordination numbers. Therefore, it made sense in this initial work to use a discriminator function that measured the difference in hydrophobic coordination number between **G**^*N*^ and **G**^T^; below, we explore a much more transferable graph distance measure.

To identify graph sequences that fold correctly to the target graph **G**^T^ we re-express each graph such that **G**^*i*^ = **G**^*i*–1^ + **C**^*i*^, where we refer to **C**^*i*^ as a graph-transition. The graph-transition **C**^*i*^ is viewed as an operator that updates the current contact-map **G**^*i*^ to give a new contact-map **G**^*i*+1^. In our previous work, each **C**^*i*^ is a triplet of integers, such that **C**^*i*^ = (*k*, *m*, Δ) where (*k*, *m*) are two residue indices and Δ = ± 1 defines whether the contact *G*_*km*_ should be formed (Δ = +1) or broken (Δ = −1). By construction, this definition of graph-transitions means that consecutive graphs must be accessible from each other by one single contact-map change.

For a given starting graph **G**^0^, application of a sequence of graph-transitions [**C**^1^, **C**^2^,..., **C**^*N*^] results in a final contact-map **G**^*N*^ that may then be compared to the target graph **G**^T^. The identification of successful folding paths in contact-map space can then be cast as a discrete optimization problem in which one seeks to find a sequence [**C**^1^, **C**^2^,..., **C**^*N*^] that yields **G**^T^. To address this optimization challenge, we employ simulated annealing (SA), where the graph-transitions **C**^*i*^ are randomly modified at each SA iteration (subject to the condition *G*_*ij*_ ∈ [0, 1]). The updates to **C**^*i*^ enable the search through graph-sequence space, allowing identification of a set of contact-map update sequences; in other words, repeated SA optimization runs yield a range of protein-folding paths in contact-map space. Finally, we note that the target optimization function used in these previous SA runs was simply the discriminator function of our final graph with the target graph *d*(**G**^*N*^, **G**^T^).

Once a given SA run has successfully converged on a folding pathway, we subsequently back-transform the resulting graph sequence into a Cartesian-space representation describing all intermediate structures along the protein-folding path. This is achieved by performing geometry optimization of the protein structure under the action of a mixed PES built from the protein interaction PES *V* (**r**) and an added biased potential *W*(**r**, **G**), referred to as the graph restraining potential (GRP):
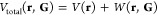
3The GRP is a pairwise interaction acting on residues:
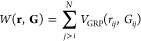
4where
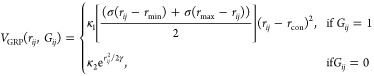
5Here, σ(*x*) is the logistic function and κ_1_ = 1 ϵ Å^–2^, κ_2_ = 6 ϵ, and γ = 6 Å^2^ are parameters determining the strength of the restraints. These were previously selected to avoid numerical or convergence issues in our initial work.^6^ The parameters *r*_min_ and *r*_max_ are the lower and upper thresholds, respectively, for residue close-contacts, and *r*_con_ = 1 2(*r*_min_ + *r*_max_) is the midpoint inter-residue distance used as a representative target value for contact-distances. Overall, eq 5 acts to enforce the contact-map **G** on the set of coordinates **r**, with the first term acting to maintain contacts for which *G*_*ij*_ = 1, and the second term acting to keep apart residues with *G*_*ij*_ = 0. As such, geometry optimization under *V*_total_(**r**, **G**) generates a protein structure that is consistent with the input contact-map **G**.

After GRP back-mapping for an intermediate structure, a final energy minimization is performed under the physical interaction potential *V* (**r**) in order to ensure that a *physical* intermediate is generated. Subsequently, NEB calculations are performed between consecutive structures in the protein-folding pathway. The energy barriers found from the NEB calculations, along with knowledge of the relative energies of intermediate protein structures, can then be used to assess the thermodynamic and kinetic plausibility of each path. As we demonstrated previously, this analysis ultimately reflects the likelihood of finding similar pathways through brute force MD simulations.

To verify our methodology, we used the Fréchet distance as a metric on paths, which is designed to respect time order but not necessarily the time-step size itself.^21,22^ The Fréchet distances between the set of paths generated by GDS and a set of trajectories obtained by direct MD simulations were used to confirm that GDS paths that are “close” to MD paths did indeed correspond to lower-energy folding pathways; in other words, this analysis confirmed that ranking GDS paths based on their energetic properties offers a route to identifying physically plausible folding mechanisms of the same type as would be generated by MD.

### 2.3 Challenges with the Initial Methodology

Although our previous methodology was sufficient to generate protein folding pathways showcasing the hydrophobic collapse for model proteins with up to *N* = 34 residues, we have identified three key problems that must be addressed before moving forward to employ GDS in studying larger, more complex proteins such as protein L/G. Here, we describe these three challenges before implementing algorithmic updates to address these.

#### 2.3.1 Combinatorial Growth of Graph-Sequence Search Space

First, we note that our initial restriction on graph-sequence updates - specifically, requiring graph-transitions to be single-contact formation/breaking events - will inevitably require an increasingly long graph-sequence search space as the size of the target protein increases. Furthermore, many protein folding events may involve multiple cooperative contacts,^23^ rather than steps involving single inter-residue contact changes. Additionally, when performing structural back-mapping as described above, we commonly find that the contact-map differences between adjacent folding intermediates (generated after optimization on the physical interaction PES) are much more extensive than single contact changes. Given the role of cooperativity, as well as the inevitable appearance of cooperative contact changes observed during structure back-mapping, it would be expected to be advantageous to modify GDS to enable graph-transitions that contain multiple contact updates. As well as better representing the physical sequence of contact updates along a folding path, we also expect that this update would enable shorter overall graph-sequences to be used in representing folding paths.

In our previous methodology, a graph-sequence update during SA optimization replaced a graph-transition **C**^*i*^ with another possible sequence update. However, when allowing for higher-order contact-map changes, as suggested above in accounting for cooperativity, the number of *possible* graph-transitions begins to grow dramatically as the size of the target protein grows. For example, allowing *any* graph-transition that involves six residues simultaneously would require  possible moves to be defined and available during SA optimization. This in turn, for a protein with *N* residues, would mean there could be up to  possible graph-transitions options; as a result the search space explored during SA optimization would grow dramatically with protein size. Furthermore, for any given protein contact-map, it is inevitable that only a subset of all possible contact-map updates would be allowed based on the restraint *G*_*ij*_ ∈ [0, 1]. If we expand our set of possible graph-transitions to account for cooperativity, the number of possible moves at each iteration will outgrow the number of acceptable moves, further slowing down the search process by demanding more SA iterations.

To summarize, single-contact graph updates, as employed in our original GDS approach, have the disadvantage of poorly capturing expected physical changes along protein folding paths - yet expanding the set of allowed contact-map updates to account for cooperativity inevitably impacts efficiency of global optimization.

#### 2.3.2 Poor Distance Metrics in Contact-Map Space

In our initial implementation of GDS, we employed a simple Hamming distance metric to assess the difference between the contact-map produced by a graph-sequence, **G**^*N*^ and the target contact-map **G**^T^. The Hamming distance metric employed was:
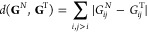
6In other words, this metric simply counts the number of different contact-map entries to give a measure of predicted structural difference.

**Figure 3:**
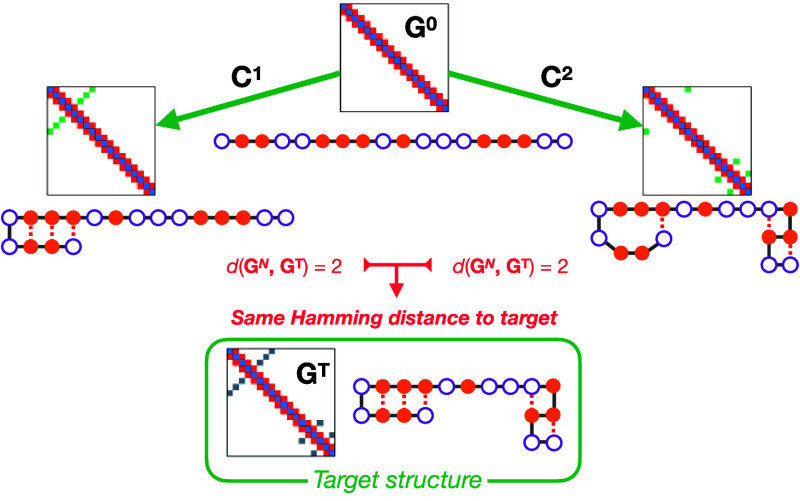
Both moves **C**^1^ and **C**^2^ correspond to only three contact-map changes relative to the initial structure of **G**^0^. Furthermore, both resulting structures from these different updates are determined to be equidistant from the target folded structure **G**^T^. However, upon inspection, the resulting structure from **C**^2^ is much closer to the target structure, and involves a much more complex structural change than **C**^1^. In fact, when back-mapped and geometry-optimized on the true interaction PES, we might expect the **C**^2^-proposed structure to readily form the target structure, whereas this would not necessarily be the case for the structure generated by **C**^1^.

In practice, the use of this simple Hamming metric introduces a significant challenge in interpreting true “distances” between the protein structures represented by contact maps, particularly once they undergo geometry optimization on the true physical PES of the system. To understand why, we consider a GDS calculation for a minimalist model in Figure 3. In this example, we consider a representative contact-map update in which two inter-residue contacts are formed as a result of a single graph-transition. However, as described above, the process of back-mapping and geometry optimization can lead to very different protein structures when optimized in Cartesian coordinates on the physical interaction PES, even though the original Hamming metric of eq 6 would failed to identify any difference between the expected structures. In other words, the Hamming metric is not a good discriminator function to employ in our SA simulations; protein structures that are predicted to be “close” in contact-map space can instead very different once transformed into Cartesian space.

A possible solution to this issue would be to perform a graph-to-structure reconstruction and geometry optimization at every single iteration of our SA runs, subsequently using a “real-space” metric such as TM-Score^24^ as the basis for an improved discriminator function. Unfortunately, this approach would be too computationally demanding to employ during SA updates; additionally, this strategy is not in the spirit of a high-throughput folding-path generation algorithm.

An alternative route is to identify a new discriminator function that *only* operates on contact-maps, but which better reflects the dissimilarity one would find if comparing reconstructed Cartesian-space protein structures.

#### 2.3.3 Failed Structure Reconstruction and Nonphysical Contact Maps

The graph representation of a protein structure effectively defines a set of distance restraints that the Cartesian-space protein structure must satisfy. Unfortunately, it is possible (for example, as an output of a GDS SA optimization) to generate a graph that does not correspond to a Cartesian structure due to violation of geometric restraints like the triangle inequality. Furthermore, even if a graph is defined such that it corresponds to Cartesian-space structure without violating geometric constraints, this does not guarantee that back-transformation is straightforward; for example, when using our artificial GRP (eq 5), we often encounter numerical issues due to high-energy steric clashes within the structure. Our previous work focused on relatively small structures, where these issues did not manifest themselves frequently; for larger protein structures, the frequency of failed structure reconstruction becomes too high to ignore such artifacts.

We note that prior studies exist in the literature on verifying whether or not a set of distance restraints is compatible with a three-dimensional structure, or to sample structures that satisfy a set of distance restraints.^25−27^ These methods are, in principle, suitable for rejecting proposed moves in our GDS simulations. However, such approaches would incur a heavy computational cost for each restraint validation and structure reconstruction - a problem that would only increase as larger proteins and longer graph-sequences are studied.

Clearly, we want to avoid methods that add too much computational overhead to our GDS SA protocol. Therefore, we will reformulate our strategy to avoid repeated checks of whether a proposed graph can be embedded in Cartesian space. As described below, our preferred approach is to quickly identify *physical* contact-maps that are minimally perturbed from any identified *nonphysical* contact-map. We note that this is a similar strategy as previously employed when mapping between internal coordinates and Cartesian coordinates,^28−30^ seeking to optimize within a physically realizable space (i.e., Cartesian coordinates) rather than verify whether the set of internal coordinates are internally incompatible.

### 2.4 Building a Better Algorithm

From the descriptions above, it is clear that there remain important algorithmic challenges that need to be addressed to transform our original GDS approach into a strategy capable of modeling larger, more realistic protein-folding problems. Here, we describe the new developments that we propose to address these problems. Later, we demonstrate the impact of these new capabilities in identifying multiple folding paths for the L/G protein.

#### 2.4.1 A Better Distance Metric: Shortest Contact Hops

First, we propose a new metric to evaluate the similarity of contact maps while also better representing structural similarity in Cartesian-coordinate space.

To motivate this new metric, it is useful to first understand how additional, nonlocal topological information can be extracted from a contact map. Clearly, the (*i*, *j*) entry of a contact map describes whether or not residues (*i*, *j*) are within the 8 Å threshold. Now, suppose (*A*, *B*) are two structures to be compared using their contact maps (**G**^*A*^, **G**^*B*^). We can also assign distance matrices to (*A*, *B*), denoted (**D**^*A*^, **D**^*B*^), noting that the distances satisfy the contact map restraints. Using the distance matrices (**D**^*A*^, **D**^*B*^) we can clearly calculate similarity measures that have been explored previously;^31^ however, we note that the transformation to real-space distance matrices introduces unacceptable computational burden to a GDS search. Instead, we demand a similarity measure that is based on contact maps alone,

To deliver this, we note that contact maps contain more information that simple (*i*, *j*) inter-residue contacts, and instead encode some information about the distance matrix. If two residues are in contact, the inter-residue distance is clearly known to be less than 8 Å; this provides additional information on the distances between residues which are in contact with the original bound pair. If a third residue is in contact with just one of the contact-paired residues, it necessarily has a distance in the range 8–16 Å with the other residue in the original bound pair. As such, the number of intermediate contacts between two residues offers an upper bound for the distance between them. In this sense, the *shortest path* between any two residues in the contact-map representation provides further nonlocal information about the inter-residue distances. In other words, comparing the topology of contact-maps, particularly the connectivity, better informs the geometric differences that would be expected in Cartesian-coordinate space.

To capture this nonlocal geometric information in a distance metric based only on contact maps, we propose here a new metric based on difference between the *shortest hop* matrices for two contact maps, (**S**^*A*^, **S**^*B*^). Here, each (*i*, *j*) entry represent the shortest number of contact-map hops required to traverse from residue *i* to residue *j*. However, we also seek to ensure that the distance function is more strongly weighted for differences in short contacts, reflecting the primary importance of inter-residue contacts in stabilizing protein structure; this a common theme found in several previous protein structure comparison methods.^24,32^ These considerations inspired the following contact-map-based distance metric:
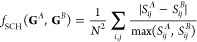
7where the denominator naturally offers lower weighting to differences associated with larger pairwise distances. This discriminator function can be shown to satisfy the triangle inequality and can be readily evaluated directly from contact maps (**G**^*A*^, **G**^*B*^) using methods such as Seidel’s algorithm.^33^ In the following, we refer to this metric as the shortest-contact-hop (SCH) metric.

#### 2.4.2 Bit Flipping Updates

Our previous methodology required prior definition of a set of graph-transitions that could be used to construct folding paths during SA optimization. As noted above, this approach leads to rapid growth of possible elementary transitions in our GDS SA protocol if one seeks to account for cooperative updates. To address this, we propose here to instead perturb a given graph sequence by “bit flipping” the contact map of one particular graph in a graph sequence [**G**^0^, **G**^1^,..., **G**^*N*^] rather than change a particular graph-transition **C**^*i*^. Here, the key idea is that any of the large number of possible contact-map changes that could be defined for a given *N*-bead protein can be written as sums of single bit-flips (i.e., changes of a single contact-map entry). As such, rather than using a large set of possible contact-map updates, we instead employ single “bit flips”; as shown later, this provide benefits such as improved SA acceptance probability and better folding-space exploration, while keeping the graph-sequences the same length.

In this framework, a perturbation to our graph sequence [**G**^0^, **G**^1^,..., **G**^*N*^] is encoded in a triplet of integers (*t*, *k*, *m*) where *t* is the selected graph to update, and (*k*, *m*) is the contact-map entry being formed/broken (between the *k*th and *m*th residue). In other words, the entry (*k*, *m*) is then flipped from a 0 to 1 or *vice versa*. In this scheme, because every graph-update is just a bit flip, we circumvent the combinatorial growth associated with choosing a move from every possible combination of *N*-bead updates.

This approach represents a similar trade-off to typical Monte Carlo (MC) move proposals. By narrowing down the range of possible Monte Carlo moves, it is generally more likely to generate accepted moves but at the cost of demanding more single moves to move across search-space. However, as described above, the combinatorial growth in potential contact-map updates for larger proteins and more-complex contact-map updates means that the single bit flip framework is much more efficient. Furthermore, as we show below, this approach does indeed enable generation of physically realizable folding paths.

#### 2.4.3 Reconstruction with Nonphysical Contact-Map Corrections

During SA run, as the sequence of graph moves [**G**^0^, **G**^1^,..., **G**^*N*^] approaches the target **G**^T^, it is possible that the intermediate graphs do not result in a successful back-transformation and geometry optimization. In the worst cases, the graphs can even encode nonphysical protein structures, with contact restraints that simply cannot be satisfied. As described above, instead of updating our SA protocol to ensure generation of physically accessible graphs by continually back-mapping to Cartesian space, we instead adopt a scheme that seeks to correct nonphysical structures such that the back-mapping to Cartesian space produces a “nearby” physical protein structure.

Here, we introduce two steps that significantly improve the stability of structure reconstruction, while at the same time minimizing the number of nonphysical protein structures that are generated. First, we modified the functional form of the GRP used in structural back-mapping. In our original GRP, parametrizing the repulsive force was often found to be difficult because the combination of multiple structural restraints, which may geometrically violate each other, can introduce significant steric clashes that are difficult to resolve by simply increasing the repulsive forces in the nonbonded part of the GRP. Here, we propose to instead replace our original GRP function with harmonic forces that only act when the required structural constraints encoded in the contact map are violated, as follows:
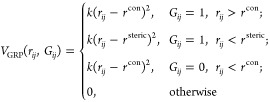
8In eq 8, we only require a single harmonic strength parameter, *k* = 100 ϵ Å^–2^, which is chosen to be quite high to correctly enforce structural constraints. We note that *r*^con^ is the threshold distance at which inter-residue contacts are considered to be formed, and *r*^steric^ = 2 Å is chosen to avoid steric clashes between residues.

In addition to updating the functional form of the GRP, we also modified the protein structure optimization strategy used to generate Cartesian coordinates. Specifically, rather than simply performing continuous optimization under the GRP, we additionally employ a Metropolis-Hastings MC procedure previously used by Domany and co-workers.^34^ This method is comparable to the crank-shaft moves performed during polymer simulations on a lattice, albeit modified to work on off-lattice models such as those of interest here. Here, MC moves are performed by selecting a bead (residue) and subsequently performing a rotation about the axes connecting to its neighbors. Such a move is rejected if a steric clash occurs, but also if it fails the Metropolis update condition using the GRP as a potential energy function. Such moves, combined with the steric clash criterion, ensure that predominantly physically sensible protein structures are generated; in turn this ensures that the generation of Cartesian coordinates for intermediate structures along the folding path is much less prone to geometry optimization failures, and the back-mapping procedure is more strongly constrained to generate relevant low-energy structures.

## 3 Results and Discussion

In the previous sections, we have highlighted three algorithmic challenges that we have identified in seeking to apply GDS to fold larger protein structures. In this section, we will begin by first validating these improvements, paying particular attention to quantifying the impact of the new SCH metric and more robust back-transformation.

Subsequently, and most importantly, we seek to validate our entire simulation protocol by identifying the two mechanisms associated with folding of the protein L/G. Here, we use our improved GDS approach to generate a contact-map folding-path ensemble from which we can clearly identify the two alternative folding mechanisms reported using previous MD simulations. This specific example, representing a larger protein structure than previously studied with GDS, in addition to multiple folding pathways, offers a strong test of our GDS strategy. As such, the success reported below supports the future expansion of this approach to fully atomistic simulations.

### 3.1 Validating the SCH Metric

We begin by assessing the use of our new SCH metric in quantifying the difference between protein structures based solely on their contact-maps. As a reminder, we anticipate that the new SCH metric should better represent the Cartesian-space structural difference between two protein structures, while *only* using contact-map input information.

To assess the SCH metric, GDS simulations are not necessary. Instead, it is sufficient to compare any protein structure pairs, regardless of their origin. Here, we use the predicted structures of three distinct proteins that were studied in the *critical assessment of protein structure prediction* (CASP14) exercise.^35^ The proteins selected - T1027 (168 residues), T1035 (102 residues), and T1040 (130 residues) - were chosen as their sizes are representative of typical single-chain proteins.^36^

To quantify the extent to which the SCH metric can capture Cartesian-space structural differences, we compare it against both the Hamming metric used in our previous work (eq 6) and the well-known template modeling (TM) score.^24^ The TM-score is one of the more common similarity metrics used in protein structure comparison, and spans the range TM ∈ [0, 1], where a score of TM = 1 indicates a perfect structural match and TM = 0 indicates no similarity. TM values above 0.5 generally suggest that two compared protein structure have the same overall fold, whereas scores below 0.17 indicate random similarity.^37^ The TM-score accounts for both the length of the proteins and the distance between corresponding residues, providing a length-independent measure that emphasizes overall structural topology rather than local deviations. This makes the TM-score a more reliable and stable metric compared to the root-mean-square deviation (RMSD), especially when comparing proteins of varying lengths. As such, it is widely used in structural bioinformatics, and competitions like CASP, to assess and rank the quality of protein structure predictions. However, we note that the TM-score is evaluated from knowledge of the Cartesian coordinates of all residues in the proteins, and we have already noted that this approach is not compatible with our GDS strategy.

In comparing the SCH and Hamming metrics, a key measure is the extent to which the metric is correlated with the TM score. Given that the TM score reflects protein similarity in Cartesian structural space, we ideally seek a contact-map-based metric that similarly exhibits strong correlation with the TM score. Such correlation would indicate that the contact-map metric correctly captures real-space structural similarity, with the advantage of not actually requiring real-space structures. To assess correlation here, we use the Spearman rank-order correlation coefficient, a nonparametric measure that assesses the strength and direction of the association between two ranked variables.^38^ Unlike Pearson’s correlation, which measures linear relationships, Spearman’s rank-order correlation evaluates monotonic relationships, making it suitable to measure the strength of the correspondence between the TM-score and the contact-map metrics. The Spearman coefficient ρ ranges from −1 to 1, with ρ = 1 indicating perfect positive monotonic relationship, ρ = −1 indicating perfect negative monotonic relationship, and ρ = 0 indicating no monotonic relationship.

**Figure 4:**
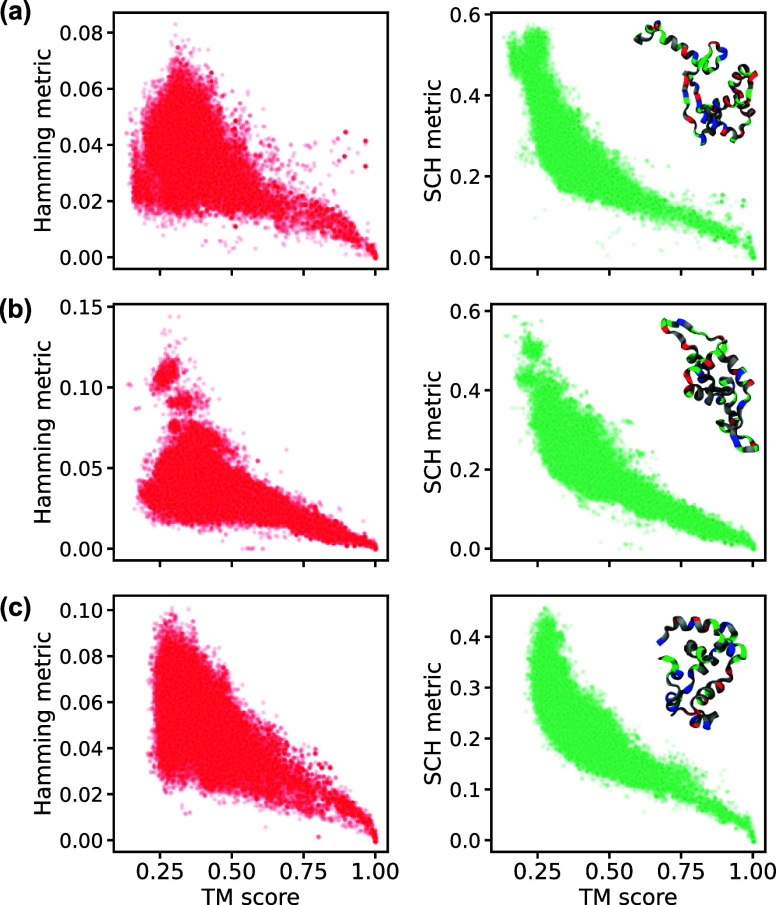
Scatter plots illustrating correlation between TM-score and either Hamming score (left-column, red) or SCH metric (right-column, green). Results are shown for three proteins from CASP14: (a) T1040, (b) T1035, and (c) T1027.

The results of the comparison of SCH, Hamming and TM-score are shown in Figure 4. The Spearman rank-order correlation coefficient for Hamming Metric against the TM-Score was −0.34, −0.65, −0.59 for the proteins T1040, T1035 and T1027, respectively. In contrast, our new SCH metric performed much better, with Spearman coefficients of −0.78, −0.84, and −0.88. Visually, (Figure 4) one can see where the improvement comes from when the TM-Score is below 0.5 the Hamming metric starts to fail to represent the structural similarity, corresponding to the regime for which the fold is likely to be of a different overall classification.^37^

### 3.2 Validation of Improved Reconstruction Algorithm

Before attempting to generate candidate folding pathways for the protein L/G, we first sought to validate the new back-mapping algorithm described above. To do so, we generated a database of potential protein-folding intermediate structures for protein L/G. First, the global minimum of L/G on the BLN PES was determined using SA global optimization with restarts. Here, a velocity-Verlet integrator was used to update the protein structure, using an Anderson thermostat. A time step of 10^–3^ tu was used and a collision frequency of 10^–3^. Each SA run started with an MD run of 10 tu, starting from a temperature of 10 ϵ, which was subsequently halved ten times and further simulated for 10 tu at each temperature. Finally, the structure was geometry-optimized using L-BFGS^39^ and subsequently stored in a database. Once this structure was recorded in the database, it seeded another SA run as described above; this restart process was repeated until the current best-guess for the global minimum did not change for 20 such repeats. To further verify that the global minimum was located, we performed further SA optimizations starting from this global minima, but with a broad range of starting temperatures; again, the best-guess global minimum did not change further. Furthermore, visual inspection of the structure in comparison with the previous work of Head-Gordon and co-workers confirmed a match. Overall, this search procedure generated a database of 254 unique local minima for the L/G protein, and these were subsequently used to assess the impact of changes to our reconstruction algorithm.

We tested the three proposed reconstruction methods - namely the original GRP-optimization strategy, optimization under the new GRP potential, and the Dormany crankshaft MC refinement - for the L/G protein system. Here, we sought to investigate two related performance criteria in reconstructing Cartesian-space protein structures from contact-maps, specifically: (i) the distance (or similarity) to the target contact-map for which a Cartesian protein structure is sought, and (ii) the impact of the starting configuration for structural back-mapping.

To enable this comparison we used the database of local minima (described above) as initial configurations. Following calculation of the initial contact-map for each structure, we subsequently simulated random bit-flip moves to modify these contact-maps; this is essentially the same process as used to generate folding intermediates in our GDS optimization strategy, and is known to generate a mixture of physical and nonphysical contact maps as targets for structure reconstruction. To further break down the impact of physical and nonphysical target contact-maps on our reconstruction procedures, we also perform a second test in which a short, high-temperature MD trajectory is initiated from a starting configuration before being subsequently subjected to geometry optimization under the BLN PES. The contact map of the resulting configuration - which, by construction, represents a physically sensible contact-map - is then used as the reconstruction target for further comparison of the three reconstruction strategies.

**Figure 5:**
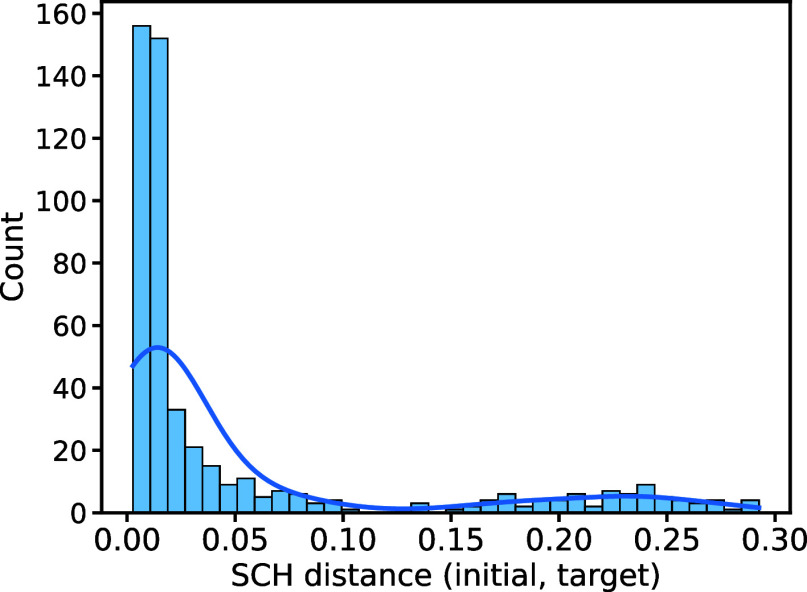
Histogram (and associated kernel density estimation [KDE]) of SCH differences between the starting contact map and the target contact map.

To assess the different reconstruction methods, we begin by randomly selecting a structure from the database of local minima. The corresponding contact-map is calculated, then subjected to a sequence of five random bit-flips; the resulting contact-map is then considered as a new target for reconstruction. As shown in Figure 5, the target contact-maps generated by this strategy span a broad range of distances from the initial graph. We subsequently use the three proposed reconstruction methods, namely the gradient-based minimization of both the old and new GRP, and the Dormany crankshaft updates, to reconstruct Cartesian-space protein structures from the target contact-maps. This procedure was repeated 500 times to give a broad range of contact-maps to help evaluate the different reconstruction strategies.

For each of the three different reconstruction methods, Table 1 presents the percentage of calculations that exhibited numerical instabilities or failures. Furthermore, we also report the average distance between the final target contact-map and the correct target contact-map for the 500 samples, as measured using our SCH Metric.

**Table 1:** Percentage of Numerical Failures Using Three Different Reconstruction Procedures, and the Reconstructed Structure’s Average SCH-Metric Distance to the Target Contact Map

reconstruction method	% numerical failures	mean distance to target
original GRP optimization	85	0.066
new GRP optimization	0	0.065
crankshaft method	0	0.065

From Table 1 it is clear that optimization under the original GRP functional form is highly susceptible to numerical errors for this complex protein reconstruction problem. As noted above, this is primarily a result of the challenges in correctly treating repulsion between nonbonded residue pairs. In contrast, and somewhat surprisingly, the two new strategies proposed here perform equally well, and appear to completely eliminate the numerical instabilities that were prevalent in our initial GRP functional form. It is clear that the original GRP-based optimization strategy used in our initial report should now be replaced with one of the new approaches described here. Upon first look, it seems that minimizing the new GRP function and the Domany crankshaft reconstruction approaches both seem equally well-suited to the problem at hand.

**Figure 6:**
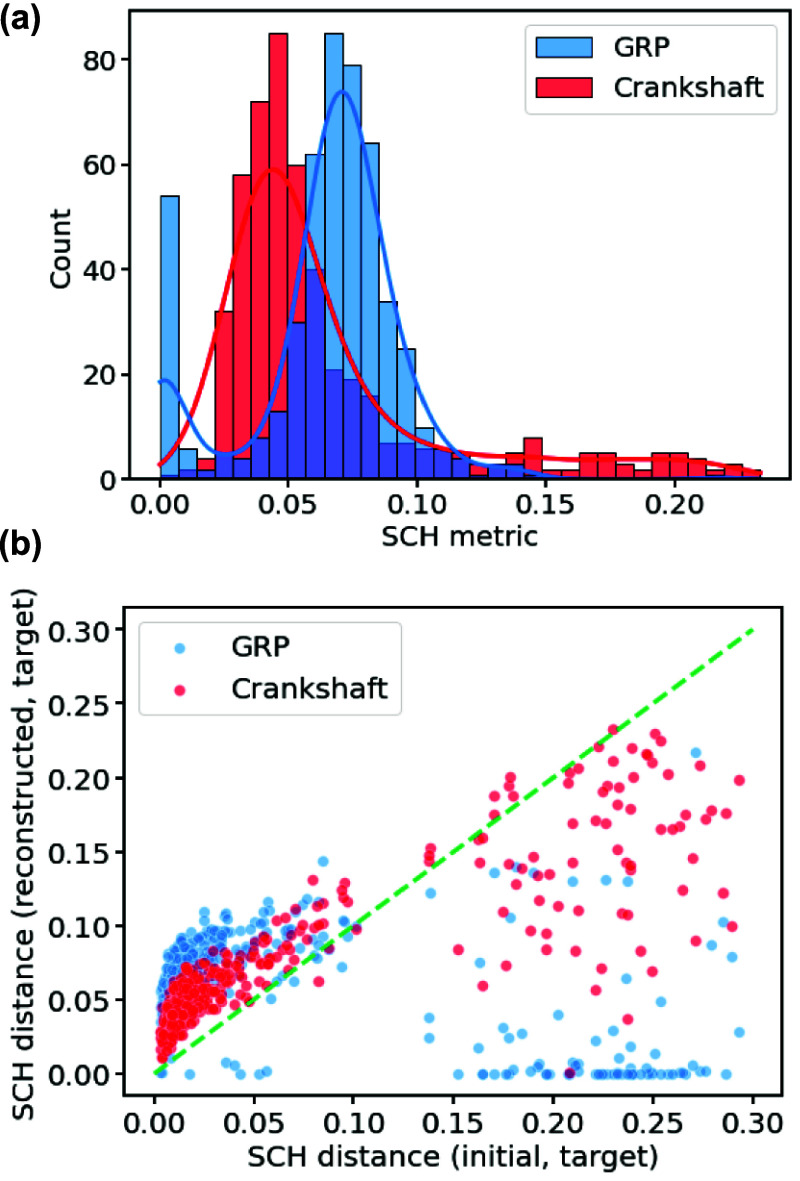
Summary of reconstruction methods. (a) Distribution (shown as both histogram and KDE plots) of contact-map differences between our reconstructed structures and the target contact-map using either optimization under new GRP or Crankshaft moves. (b) Scatter plot showing reconstruction error against the difference between the target contact map and the initial structure. The green dashed line is the diagonal, shown to highlight data trends.

To further analyze the difference between the two new reconstruction methods, we consider the impact of the distance between the initial and target contact-maps on the contact-map obtained from reconstruction. Here, we find that the gradient-based GRP optimization and Dormany crankshaft procedure differ greatly in performance. Figure 6 shows that the crankshaft-move strategy often struggles to deal with large proposed structural changes; specifically, when the target contact-map is far from the contact-map chosen as the starting point for refinement, the crankshaft strategy often struggles to reduce the distance to the target contact-map. Overall, this is perhaps expected, given that the crankshaft moves are local moves (and limited to a finite number of MC steps), whereas the gradient-based optimization updates the whole protein structure simultaneously. Another observation is that there is a partitioning of the distribution for the GRP-minimized structures, with one of the distributions distributed very close to zero-distance from the target contact-map.

The source of this bimodal distribution becomes clear when performing similar test using target contact maps that *only* represent physically accessible contact-maps. To test the reconstruction methods using only physical structures, we perform an identical experiment as described above, but we modified the procedure for proposing a contact-map change to employ short, high temperature MD trajectories (instead of bit-flips). Specifically, we ran velocity-Verlet integration with an Anderson thermostat set to a high temperature of 1.0 ϵ. A time step of 10^–3^ tu was used and a collision frequency of 10^–3^. The dynamics were run for 10 tu before L-BFGS was used for geometry optimization; the resulting protein structure was subsequently used as a reconstruction target.

**Figure 7:**
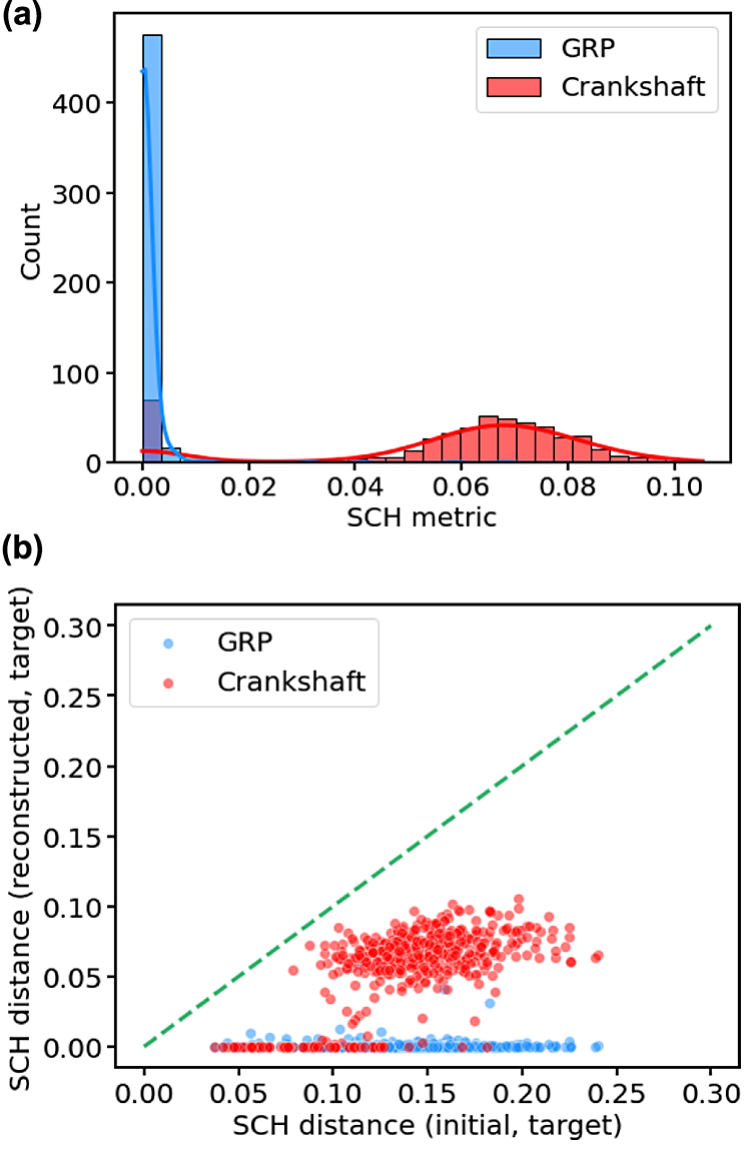
Reconstruction performance when targeting only physical contact-maps. (a) Distribution (shown as both histogram and KDE plots) of contact-map differences between our reconstructed structures and the target contact-map using either optimization under new GRP or Crankshaft moves. (b) Scatter plot showing reconstruction error against the difference between the target contact map and the initial structure. The green dashed line is the diagonal, shown to highlight data trends.

The results of this additional test (Figure 7) provide further insight into the nature of the reconstruction methods. We find that, for both methods, the distance to the target is effectively reduced, especially in the case of the gradient-based GRP optimization, which is found to be capable of reproducing the target contact-map in the majority of cases. Furthermore, Figure 7 also serves to explain the bimodality in the results of Figure 6; specifically, it is clear that the data clustered around very small shortest-hop distances (where the reconstruction was essentially perfect) corresponded to physical contact maps, whereas the larger distances comprised nonphysical maps.

### 3.3 Using GDS to Identify Folding Paths in Protein L/G

Having shown how the algorithm improvements developed here - namely better optimization metrics, better folding path exploration moves, and better structural reconstruction - can be independently assessed, the final objective of this articles is to demonstrate that these collective improvements enable our GDS strategy to tackle much more complex protein-folding problems than previously accessible. As such, we turn to consider the identification of folding paths for the L/G protein, with particular emphasis on assessing whether GDS is now capable of identifying the two distinct folding paths that are accessible in this system.

To proceed, we follow the procedure that was outlined in our previous work. First, we will build a data set of proposed folding paths using GDS. Subsequently, we will energetically filter to select a small number of folding paths that are deemed representative of the full path-ensemble. Finally, we will cluster these folding paths in an attempt to identify common structural signatures of alternative folding paths.

Given that the L/G protein contains 56 residues, with 295 possible non-neighbor contacts (assuming neighbors are separated by fewer than three peptide bonds), we highlight a final further update to GDS that dramatically helps in improving the efficiency of our folding-path search. We note that, if we were to restrict graph-sequences such that a predefined number of contact-map changes was enforced for adjacent path intermediates, this may ultimately demand an unnecessarily long folding path. For example, if we limited ourselves to generating paths that could only change a maximum of two contact-map elements at each elementary step, then identifying a folding sequence in the contact-map space comprising 2^295^ elements might require an extremely long allowed path and hence a challenging optimization problem. Furthermore, as highlighted above, the number of contacts changed is not necessarily indicative of the significance of a given conformational change.

Instead, we chose to perform our GDS simulations with minimal restrictions on the relationship between the adjacent contact-maps. We do, however, restrict the final and starting contact map, **G**^*I*^ and **G**^*F*^, to be the contact maps of the initially unfolded protein and the folded structure, respectively, using GDS to optimize the intermediate contact maps. In order to drive our GDS searches toward sequences of contact maps that are more likely to be possess physically relevant intermediate structures, we additionally modify the GDS SA optimization function to the following:
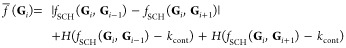
9Here, *f*_SCH_ is the SCH metric (eq 7), *H* is the Heaviside step function, and *k*_cont_ = 0.15. Discrete optimization of the folding path under this function acts to steer the search away from violation of connectivity criteria; the first term tries to make sure an intermediate contact map **G**_*i*_ is approximately equidistant between the previous and next contact map while the later terms punishes a move that exceeds some threshold *k*_cont_. The choice of *k*_cont_ is based on Figure 4, where a value of *d* = 0.15 typically corresponds to a TM-score of around 0.5, a value that is usually indicative of a change in folding topology.^37^

Using this improved GDS strategy, we generate 2877 independent folding paths that were constrained to contain three intermediates (so total sequence-lengths of five). Each path was initiated such that the starting contact-map corresponded to a fully unfolded structure and the final contact-map corresponded to the folded native state. The SCH Metric between the fully unfolded structure and the final structure was 0.53. Since we expect each transition to be around the 0.15 difference mark, we chose to have 4 transitions. If fewer transitions were used, due to the metric nature of the SCH function, we could not guarantee that each consecutive difference would be 0.15 or less. Each GDS optimization employed 10^4^ MC updates with a linearly decreasing temperature protocol starting at *T* = 10^–3^ reduced units. Each MC update randomly selected an intermediate graph, perturbed it with at least *n*_b_ ∈ [1, 5] bit-flips chosen, and then employed optimization under the new GRP to reconstruct physically relevant protein intermediates.

To evaluate the relative importance of GDS-proposed folding paths, we used additional information from nudged elastic band (NEB) calculations^10^ between all consecutive intermediates found in each folding sequence. However, we found that initial interpolation was a significant challenge, even when using interpolation schemes such as image-dependent pair-potentials.^40^ This can be attributed to the complex nature of the protein configurations, where typically several dihedral angles must be collectively updated to provide a reasonable low-energy starting path for NEB optimization. As a result, we instead employed the freezing string method (FSM^41^), a variant of the growing string method (GSM^42^), to generate interpolated paths. Both GSM and FSM avoid generating a full initial-interpolation path by instead iteratively advancing paths from both ends of a transition. The key conceptual difference between these approaches is that GSM aims to generate the minimum energy path (MEP) while “growing” the path, and so continually optimizes images along the path; in contrast, FSM instead focuses on generating a reasonable initial starting point, so only optimizes the single “advancing” image. Because of this difference, FSM is more efficient in providing an initial interpolation, which we subsequently use in a further NEB refinement.

To advance images in FSM, we use linear synchronous transit (LST) as described in the original FSM report. However, one deviation that we do take from the original FSM strategy is to add a further condition to the image-advancement step in order to prevent generation of paths that “skip through” steric clashes. Here, we define a force threshold while advancing the nodes; if the force exceeds this threshold, the image-advancement is frozen to provide better resolution in high-energy regions.

All FSM calculations were preformed with a maximum of ten images from each end-point; in other words, we obtain paths parametrized by a maximum of 20 nodes. Each advance is capped at a RMSD of 2 Å, or stopped when the RMS force is greater than 100 ϵ Å^–1^. The same minimization protocol was used as described in the original FSM paper.^41^ Once an FSM calculation was complete, ten equidistant images where chosen to represent the final path, based on LST interpolation of the converged FSM path. These images were then used to perform a NEB optimization using the QuickMin algorithm^43^ for a maximum 10^4^ iterations. The convergence criteria demanded a RMS force threshold of 10^–4^ ϵ Å^–1^, and a maximum force of 10^–2^ ϵ Å^–1^ on each image.

#### 3.3.1 Folding Path Analysis

The folded structure of the L/G protein comprises two β-hairpins on both ends of an α-helix structure (Figure 1). The two folding pathways detected previously by Head-Gordon and co-workers were primarily characterized using two order parameters that each measure the degree of formation of one of the β-hairpins. These order parameters are defined as
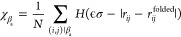
10where β_1_ and β_2_ refer to the two respective β-hairpins. The sum in eq 10 is performed over all *N* residue-pairs in the β-hairpin in question, *H* is the Heaviside function, *r*_*ij*_ and *r*_*ij*_^folded^ are the distances between beads (*i*, *j*) in a given structure and the folded structure, respectively, and we set ϵ = 0.2 to account for small fluctuations away from the folded state. We note that χ-values close to one correspond to folded configurations. To enable comparison with previous work, we use the same order parameter to characterize our GDS-generated folding paths. Specifically, we seek to confirm whether we can observe the two distinct folding paths that are characteristic of protein L/G. These two paths differ in which of the two β-hairpins forms first, and can be identified using eq 10. In one folding path, χ_β_1__ approaches a value of one, followed by χ_β_2__ approaching one; in the alternative folding path, this sequence is reversed.

The ensemble of 2877 folding paths generated by GDS are, of course, not all equally likely to be observed. As we have discussed previously, we expect that only the most energetically favorable paths will be representative of the true path-ensemble. As such, we chose to further analyze the 100 folding paths with the lowest values of the “floored” energy Δ*E*^+^ along the folding path. For the sequence of structures along the NEB converged folding path (**r**_1_, **r**_2_,..., **r**_*n*_), Δ*E*^+^ is defined by summing over all energy barriers, as follows:
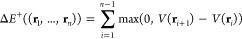
11To identify path-similarities in our 100 selected folding paths, we subsequently employed a clustering analysis. Here, we employed the discrete Fréchet metric - shown to be a useful metric in comparing MD trajectories^21^ and utilized in our previous work^6^ - on the χ_β_ order parameter description of the pathways. The pairwise Fréchet distance matrix of all paths was then used to identify similar paths using the hierarchical density-based spatial clustering of applications with noise (HDBSCAN) strategy.^44^

**Figure 8:**
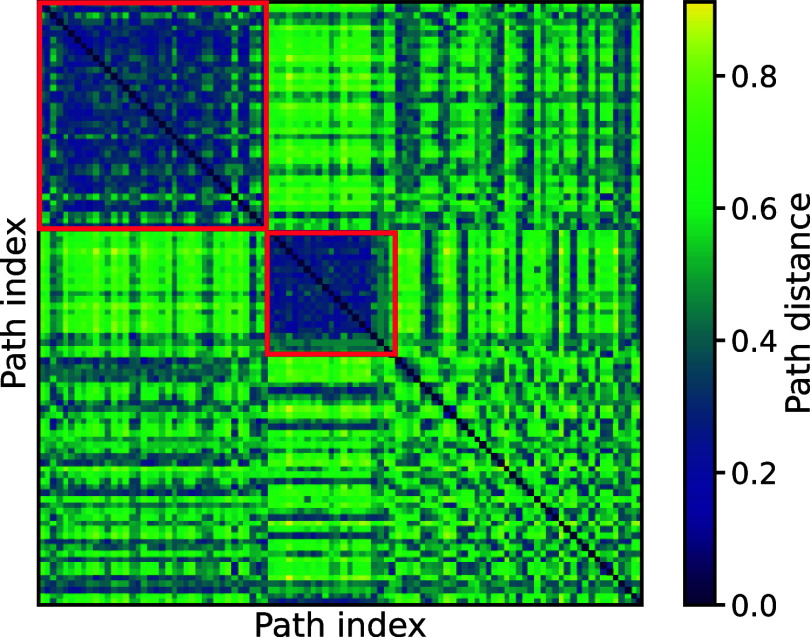
Heatmap representation of the matrix of Fréchet distances, reordered based on identified clusters. There are two, well-defined self-similar clusters of folding paths (highlighted by red boxes).

As shown in Figure 8, this analysis clearly demonstrates the emergence of two distinct clusters of folding paths that are identified as being similar according to the Fréchet distance matrix. Furthermore, the off-diagonal elements of the path-distance matrix also show that these two clusters are distinct from each other, and also distinct from the remaining set of unclustered trajectories.

**Figure 9:**
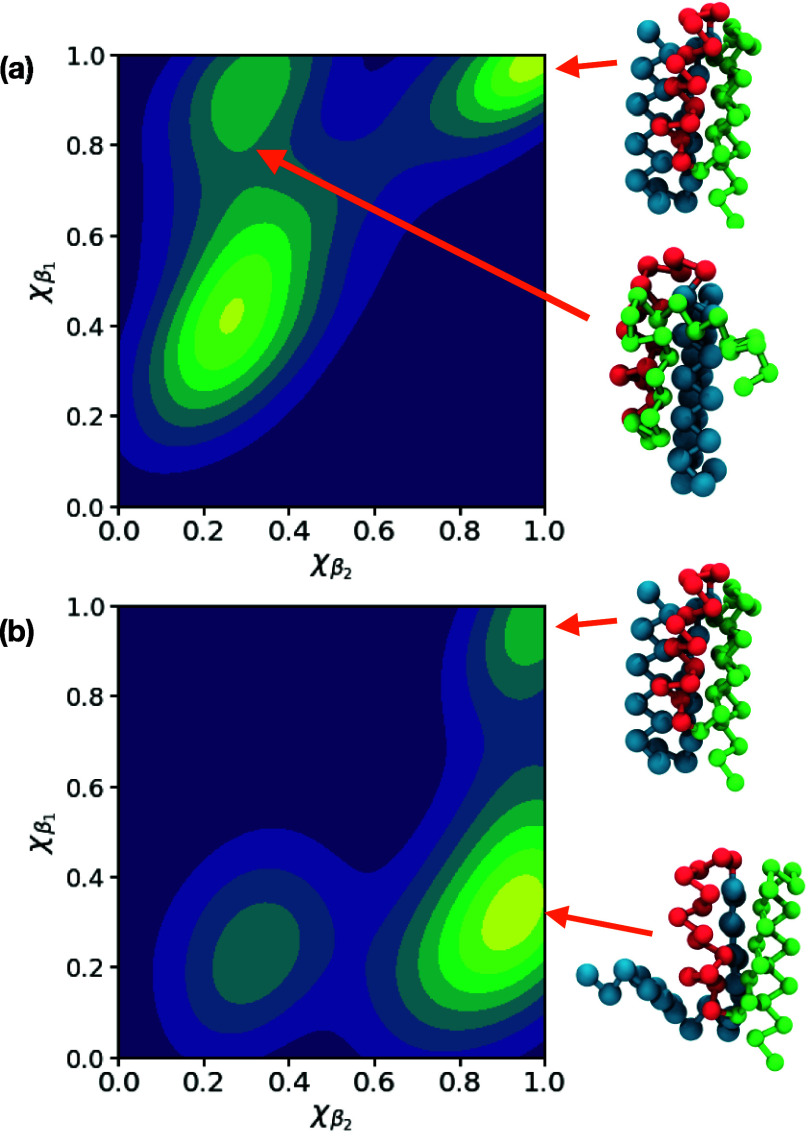
KDE plots of the intermediate structures between the unfolded regime (χ_β_1__ < 0.4 and χ_β_2__ < 0.4) and folded regime (χ_β_1__ > 0.8 and χ_β_2__ > 0.8). Panels (a) and (b) show KDE plots generated using intermediates from the two distinct clusters identified by the Frèchet distance matrix. We find that these two clusters correspond to the two folding pathways identified by Head-Gordon and co-workers. The structures on the right show representative intermediate structures, as well as the final folded structure; in each case, the beads are colored according to their secondary structure in the final state, with green and blue highlighting the formation of β-sheets and red highlighting the α-helix. These structures demonstrate that the two folding pathways correspond to different folding sequences for the two β-sheets.

To check whether these clusters do indeed correspond to the two folding pathways found in previous work, we pruned the trajectories to focus on the sections that first leave the unfolded regime (χ_β_1__, χ_β_1__ < 0.4) and ultimately lead to the folded structure (χ_β_1__, χ_β_2__ > 0.8). The resulting kernel density estimation (KDE) of the intermediate structures for the folding paths, projected onto (β_1_, β_2_) are shown in Figure 9. Satisfyingly, we find that the two clusters of folding paths generated by GDS correspond to the two folding pathways previously identified by Head-Gordon and co-workers. Specifically, we find that one pathway exhibits an increase in χ_β_1__ followed by increasing χ_β_2__, whereas the second pathway has the progress of these two swapped around.

**Figure 10:**
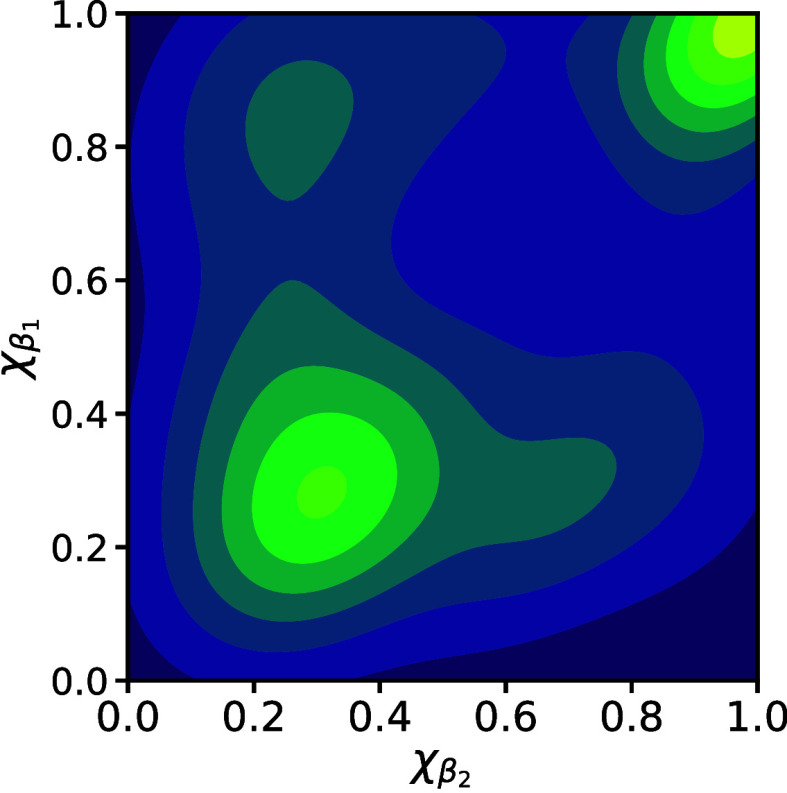
KDE plot constructed using intermediates from unclustered low-energy pathways. The density estimate appears similar to the sum of densities from the two folding-path clusters; closer inspection reveals that the sequences of intermediate exhibit “reversals”, turning back along initial paths.

Finally, it is interesting to examine the characteristics of the remaining low-energy folding paths that did not belong to the two clear clusters. Figure 10 shows the same KDE projection of the folding intermediates projected onto (β_1_, β_2_). This projection indicates that these folding paths are still similar to the clustered pathways. On closer inspection, we find that these paths involve “reversals” in which the path begins to form a particular β-hairpin, before reversing and subsequently forming the other β-strand before passing on to the folded state. Such paths are still clearly considered to be physically accessible according to the criteria employed here, but suggest that further pathway filtering based on such observations might be useful.

To summarize, we have shown how refinements and improvements of our original GDS strategy have enabled an application to study a much more complex protein-folding problem than previously accessible to our methodology, both in terms of structure complexity and folding-path heterogeneity. We emphasize that our GDS approach requires neither prior definition of order parameters or long MD trajectories, instead seeking to operate predominantly in contact-map space to accelerate determination of folding paths. The results presented here show that GDS is a rapidly evolving strategy that is growing in capability - our next target is to build on the advances reported here to apply GDS to study protein folding for fully atomistic models, and we hope to report on this application in the near-future.

## 4 Conclusions

In this article, we put forward a graph-based strategy that can identify the two distinct mechanisms involved in the folding of the protein L/G. Determining these mechanisms previously required extensive CG MD runs but, using our contact-map path-sampling approach to circumvent the time-scale problem of protein folding, we successfully identified the two possible folding paths in this system. To realize this effort, we built on our original GDS approach by tackling algorithmic challenges and in the process were able to study larger and more complex proteins. These challenges involved incorporating more complex structural changes in our optimization strategy, addressing the poor suitability of the Hamming metric as a measure of structural difference, and dealing with the numerically challenging back-transformation from contact maps to Cartesian-space structure.

The bit-flipping perturbation of a graph sequences has allowed us to better incorporate many-body graph-transitions without requiring an increasingly large set of possible graph updates. Furthermore, by addressing the deficiency of the optimization metric in contact-map space by introducing the SCH metric, we were also better equipped to identify more natural folding paths with shorter overall graph-sequence lengths. Finally, we examined alternative back-transformation methods and found a suitable strategy that works well in generating protein structures that are close to a target contact map.

The advances demonstrated here greatly increase the efficiency of our path-ensemble generation method, and have enabled application to a more challenging problem. We can now envisage a simulation setup in which postulated folding paths may be used to form folding mechanism hypotheses, combined with automated reaction-coordinate evaluation. As noted above, we also plan to study all-atom protein models in solvent environments; this will ultimately require better consideration of free-energy calculation techniques.^45−48^ It is clear that moving forward in this direction will require approaches to estimate the free-energy barriers or transition rates between contact-map-defined states that span from cheap and inaccurate heuristics for virtually screening proposed pathways to expensive and accurate methods to identify the specific mechanisms at play. Furthermore, one may need to be selective on which postulated paths should be used to perform more detailed free energy evaluations and reaction rate calculations. Balancing exploration of the path ensemble in contact-map space (which is typically very fast due to using a simple discretized structural representation) and accurate rate calculations (which is typically more expensive relative, even using barrier-based analyses such as NEB) will be an important step moving forward. However, improving both steps stand as a challenge for future work - but the results presented here already serve to highlight the promise of contact-map-based strategies for dealing with time-scale problems in molecular simulation.

## Data Availability

Data and scripts used to generate Figures 4–10 are available through the Warwick Research Archive Portal at wrap.warwick.ac.uk/186876.
